# The Effect of Cognitive Load on the Retrieval of Long-Term Memory: An fMRI Study

**DOI:** 10.3389/fnhum.2021.700146

**Published:** 2021-10-13

**Authors:** Minoo Sisakhti, Perminder S. Sachdev, Seyed Amir Hossein Batouli

**Affiliations:** ^1^Institute for Cognitive Sciences Studies, Tehran, Iran; ^2^Neuroimaging and Analysis Group, Research Center for Molecular and Cellular Imaging, Tehran University of Medical Sciences, Tehran, Iran; ^3^Centre for Healthy Brain Aging (CHeBA), School of Psychiatry, University of New South Wales, Sydney, NSW, Australia; ^4^Department of Neuroscience and Addiction Studies, School of Advanced Technologies in Medicine, Tehran University of Medical Sciences, Tehran, Iran

**Keywords:** long-term memory, visual memory, cognitive load, memory retrieval, functional MRI (fMRI)

## Abstract

One of the less well-understood aspects of memory function is the mechanism by which the brain responds to an increasing load of memory, either during encoding or retrieval. Identifying the brain structures which manage this increasing cognitive demand would enhance our knowledge of human memory. Despite numerous studies about the effect of cognitive loads on working memory processes, whether these can be applied to long-term memory processes is unclear. We asked 32 healthy young volunteers to memorize all possible details of 24 images over a 12-day period ending 2 days before the fMRI scan. The images were of 12 categories relevant to daily events, with each category including a high and a low load image. Behavioral assessments on a separate group of participants (#22) provided the average loads of the images. The participants had to retrieve these previously memorized images during the fMRI scan in 15 s, with their eyes closed. We observed seven brain structures showing the highest activation with increasing load of the retrieved images, viz. parahippocampus, cerebellum, superior lateral occipital, fusiform and lingual gyri, precuneus, and posterior cingulate gyrus. Some structures showed reduced activation when retrieving higher load images, such as the anterior cingulate, insula, and supramarginal and postcentral gyri. The findings of this study revealed that the mechanism by which a difficult-to-retrieve memory is handled is mainly by elevating the activation of the responsible brain areas and not by getting other brain regions involved, which is a help to better understand the LTM retrieval process in the human brain.

## Introduction

Memory is essential for many cognitive abilities of the human (Batouli and Sisakhti, 2019), and therefore a clear understanding of the mechanism of human memory is vital (Batouli et al., 2020). The muscles of the body work harder in a more demanding situation, such as when lifting a heavier weight; this process could be used as a metaphor to suggest what happens in our brain during a cognitive load. Cognitive demand, or as is more often called “cognitive load,” refers to the amount of information the brain simultaneously processes. In everyday life, retrieving information from memory, and particularly Long-Term Memory (LTM), in order to perform the given tasks is essential. This retrieval usually happens without intention and is able to survive at complex situations, such as when performing several tasks at once (Fischer et al., 2007). As an example, when reading a word in a sentence, its meaning comes from our LTM; however, this process is more demanding when reading verbal compounds, or when reading multiple words in a syntactic structure (McIntyre, 2007). There are reports on the retrieval process being unaffected in higher demands (e.g., concurrent task performance) (Peterson and Zill, 1986; Naveh-Benjamin et al., 2000), whereas the impairment of memory retrieval in such situations is also reported (Jacoby, 1991; Moscovitch, 1992).

There are numerous fMRI studies performed on the modulation of cognitive load in working memory (WM), but only rare studies in LTM, as briefly reviewed in below. Increasing the cognitive load in WM mostly happened by increasing the number of items (alphabets, digits, or images) to encode or retrieve, or by changing the order of items to be encoded from sequential to random, to make it more difficult. The N-back and the Sternberg Item Recognition tasks were also used for such purposes. A summary of the findings of those studies includes a significant deterioration of performance when increasing load as well as a linear activation increment in the involved brain regions (Cairo et al., 2004), the influence of aging on the brain reaction toward an increasing memory load (Bennett et al., 2013), higher involvement of middle frontal and superior parietal areas when increasing verbal WM load (Fegen et al., 2015), higher activation of the prefrontal, cerebellum, cingulate, and caudate areas in the higher auditory WM load (Leung and Alain, 2011), involvement of the cerebellum in the retrieval of high load of visual WM (Sobczak-Edmans et al., 2016), and observing an interaction between the visual WM load and feature conjunction during retrieval (Kochan et al., 2011). Based on the findings of these WM studies, we hypothesized that a mechanism by which the brain responds to a higher cognitive load is mostly by the increment of activation in the involved brain areas, and often by including additional brain regions. A review of the LTM studies in the field, as provided below, would make our hypothesis more accurate.

There was only one fMRI study on the effect of cognitive load on LTM retrieval (Zysset et al., 2001). In this work which used an event-related fMRI design, the aim was to identify the underlying neural structures of the retrieval from long and short word lists, and their comparison. It was observed that in higher load (by increasing the length of list of words), no additional brain region was involved in LTM retrieval, but the involved brain regions showed adaptations (an increased activation) to the increased demand. In addition, a greater hemodynamic change was detected in the contrast of “long minus short” in the supplementary motor area, left inferior frontal gyrus, left precuneus, left intraparietal sulcus, bilateral insula, and right thalamus. This study illustrated that the involved brain areas show an increased activation when the cognitive load of a cognitive function elevates (Zysset et al., 2001), and one study suggested that the increment of activation in a brain area under a higher load of a function shows the involvement of that brain area in the function of study (Leung et al., 2005).

As a result, our hypothesis in this work was an elevated activation in the brain areas when retrieving a higher load of information from the LTM. The available study on modulating the cognitive load in LTM (Zysset et al., 2001) assessed the memory of list of words, whereas we aim here to identify the pattern of brain functions when retrieving images from the LTM and under a varying cognitive load, which is categorized as an episodic memory.

There are candidate brain areas to be active in the retrieval of images from LTM. Hippocampus has been very often reported here (Söderlund et al., 2012; Schurgin, 2018; Takeda, 2018), and for example one study showed that consciously retrieving details from LTM is associated with a continuous activation of the hippocampus (Lara and Wallis, 2016). This area is also reported to be active when retrieving recent memories (Söderlund et al., 2012; Takeda, 2018), whereas others have challenged this finding and declared that the hippocampus is similarly engaged in either recent or remote memory retrieval (Rekkas and Constable, 2006; Söderlund et al., 2012). In addition, studying brain activity during visual LTM (e.g., faces and scenes) retrieval showed the involvement of anterior temporal areas while remembering remote memories regardless of the category (face or scene), and a more activation in the posterior areas during the retrieval of different categories (Takeda, 2018). By almost a similar paradigm, researchers investigated cortical activations during the recall of paired stimuli (word-face, and word-spatial position) to see whether retrieving from LTM yields in different cortical activations as is dissociable during perception; they found more activations of the parietal and precentral cortices for the spatial positions, and a higher activation of the left prefrontal, temporal (including fusiform gyrus) and posterior cingulate area for faces (Khader et al., 2005). In another research, a SenseCam was worn by the participants for 2 days, to collect pictures of their everyday activities (Milton et al., 2011), and later, recognition memory tasks were performed inside the MRI scanner, after 36 h and also after almost 5 months. Subjects had to distinguish between “Remember,” “Know,” and “New” pictures, as detailed previously (Milton et al., 2011). This study showed the activation of the medial temporal lobe and medial prefrontal cortex, with the activation of the hippocampus and anterior parahippocampal gyrus showing a decrement after 5 months, compared to the first time-point.

In addition to our hypothesis on an elevated activation in the brain areas involved in LTM retrieval in a higher load, we hypothesized an altered functional connectivity between those brain structures. One previous work illustrated an altered functional connectivity between the brain areas in a more demanding task (Schott et al., 2013), and it is suggested that the changes in the patterns of functional connectivity between the brain areas is a response of the brain to comply with the increased demand (Rissman et al., 2008).

## Materials and Methods

### Participants

The participants included 32 healthy young individuals (18F), with the mean age of 30.16 ± 6.4 (20–39 years old), and a minimum of 14 years of education (BSc student and above). The criteria for selection of the individuals were based on previous works (Batouli et al., 2021; Razavi et al., 2021), and in summary included: weight not over 110 KGs; not using drugs or alcohol (only based on the subjective report); consent to participate in all steps of the study; and not being claustrophobic. The following exclusion criteria were applied: any diagnosed internal/neurologic disease; long-term or current use of medications; any history of chronic headache, tinnitus, dizziness, seizure, or nausea; family history of any neurologic disease; any surgery with anesthesia; history of losing consciousness or head trauma; and any metal objects in the body, such as a pacemaker, dental brace, coronary stent, implant, or tattoo (Batouli and Sisakhti, 2020). All participants provided written informed consent, and the ethics approval was provided for this study (approval No. IR.NIMAD.REC.1396.319). These participants were regarded as the “main group.”

### Health Assessment

Each participant was examined by a physician for blood pressure, heart and respiratory rates, and a neurological examination including vision and hearing. To ensure the mental health in the participants, the Depression Anxiety Stress Scales (DASS-21) (Henry and Crawford, 2005) was administered, normalized for the Persian population (Sahebi et al., 2005).

### Memory Tests

The memory function of the participants was assessed up to 2 weeks after the fMRI exam, using the following tests: (I) Episodic Memory: Rey Auditory Verbal Learning Test (RAVLT) (Mitrushina et al., 1991), normed for the Persian language (Rezvanfard et al., 2011); (II) Working Memory: Forward and Backward Digit Span tasks, as subtests of the Wechsler Memory Scale Revised test (Wechsler, 1987). The purpose of performing these memory assessments was to ensure a healthy memory function in the participants.

### Task Design

#### Cognitive Load

A review of previous WM studies showed that the cognitive load was mostly manipulated using a limited number of approaches, such as by increasing the number of items to memorize, changing the complexity of the objects to encode, or memorizing sequential vs. random, or similar vs. different items. We used similar approaches for manipulating load in LTM, and as a result, we selected two categories of images (high load and low load) which were different in a higher number of items to memorize, a more complex episode to memorize, and the low load images being in two colors (including white) whereas high load images being in at least nine colors.

#### Images

**FIGURE 1:**
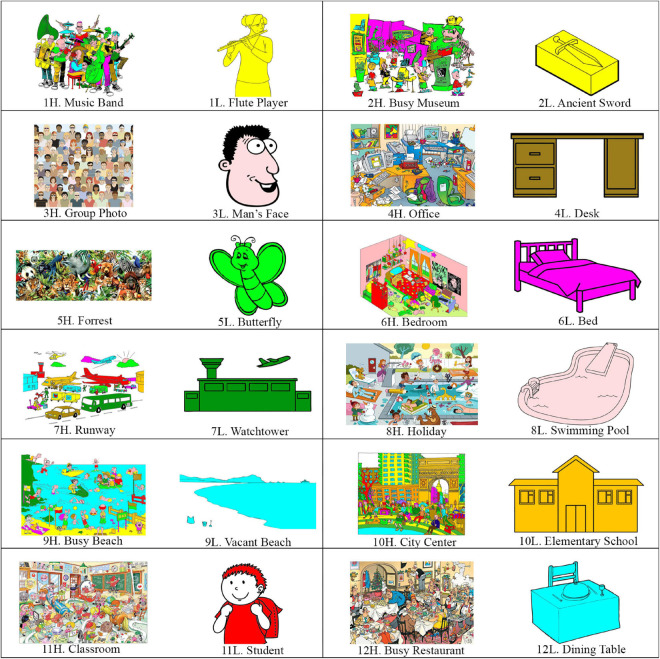
The 24 imagesused in the study. The images are in 12 categories, with each category including a high load and a low load image. The names (used as the cue) devoted to the images (a two-word name in the Persian language) are also provided.

Twelve categories of images relevant to daily events were selected, including music band, museum, human face, office desk, animals, bedroom, airport, swimming pool, beach, city center, classroom, and restaurant. Two images were selected for each category, representing the high and low load images. They were selected from Google Images, with the criteria of having good quality, being line drawing (manually colored by authors), and including a number of items in an episode relevant to the theme of the categories. We specified a two-word name to each image, which was used as the retrieval cue during the fMRI scan. The selected 24 images are illustrated in Figure 1.

#### Behavioral Assessment

A second group of participants, called behavioral group, including ten male and twelve female, all healthy and in the age range of 22–43 years (mean = 32.7 ± 6.4), were selected to score the cognitive load of the 24 images. The 24 images were randomly presented on a computer screen, and the participant looked at all images with no time limitation. In the second round of looking at the images again in a random order, the individual was asked to give a score from 1 to 10 to each image based on the amount of data that the image included and could be memorized. This score was regarded as the cognitive load of the image.

#### Encoding and Consolidation

The images were electronically sent to each individual (*via* Email, Telegram, or WhatsApp) 14 days before the fMRI test. The participants were asked to look at the images as many times as possible, until they could memorize the maximum details of each image, similar to the approach used in a previous study (Zysset et al., 2001); in addition, they were told that they will be asked of the details of the images on the scan day. The participants were volunteers from an elite community; however, to ensure their precise following of the instructions, they were checked every day by phone. Two days before the test, the participants were asked not to look at the images any further, even if they had not fully memorized them. Therefore, they had 12 days to memorize the images, and had two nights of sleep (consolidation phase) before their fMRI test.

#### fMRI Stimulus

The participants were trained about the fMRI task before entering the MRI scanner. The instructions were repeated before and during the scan. The participant was asked to retrieve an image once its name (cue) was read to him/her during the fMRI, and to mentally review (while eyes closed) all the details of it in the shortest time.

**FIGURE 2:**
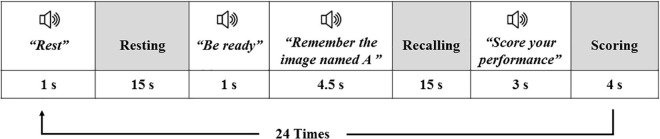
The schematic design of one trial of the fMRI task; white boxes are relevant to the auditory instructions, and the gray boxes show the periods the participant was taking rest or was performing an action. The same trial was repeated for the 24 images of the task.

There were 24 trials in the task, relevant to the 24 images, with each trial including: an auditory instruction “Rest” + 15 s of resting-state + an auditory instruction “Be ready” + an auditory instruction “remember the details of the image A” + 15 s interval (retrieval phase) + an auditory instruction “Give a score to the quality of your retrieval” + 4 s interval (giving response). The schematic design of one trial of the task is illustrated in Figure 2. The trials of different images were only different in their auditory instructions, and the high and low load trials were randomly intermixed and presented in a pseudo-randomized order. The task lasted for 17:45 min (355 fMRI volumes).

In two steps of the trial, the participant used the MR-compatible response keys: (i) if remembering all details of an image took less than 15 s, the participant pressed a particular key to inform us of the time that the retrieval finished; (ii) to give a score to the quality of the retrieval, the participant used one of the four keys to rate his/her retrieval of each image as weak, moderate, good, or excellent, which corresponded to the scores of 1–4, respectively. The participants were told to rate their retrieval as “weak” if they could not recall any details of an image or they could not remember which image was related to this cue, and the other scores (2,3,4) were relevant to the amount of details they could recall from each image.

### Imaging

The MRI machine was a Siemens 3.0 Tesla scanner (Prisma; Siemens Healthcare GmbH, Federal Republic of Germany; Production: 2016), devoted to research. Using a 64-channel head coil, the functional T2^∗^-weighted images were collected using blood oxygen level-dependent (BOLD) contrast, with 40 mT/m gradients, and by coverage of the whole head. The protocol included a single-shot, spin-echo, echoplanar imaging sequence (EPI) with the following settings: measurement = 355; TR = 3,000 ms; TE = 30 ms; flip angle = 90 degrees; voxel size = 3.0 mm × 3.0 mm × 3.0 mm; number of slices = 40. Three-dimensional T1-weighted anatomical scan was acquired before the EPI scan, using a gradient echo pulse sequence (TA = 4:12 min; TR = 1,800 ms; TE = 3.53 ms; TI = 1,100 ms; flip angle = 7 degrees; voxel size = 1.0 mm × 1.0 mm × 1.0 mm; Matrix size = 256 × 256 × 160; Averages = 1). MR-compatible headphone and response keys were used during the scan, and the headphone volume was set on a comfortable level before the scan.

### Data Analysis

#### Quality Check

The MRI data were initially checked for matrix and voxel sizes, as well as the images being right-to-left oriented. Next, a visual check was performed in order to spot possible macroscopic artifacts, vibration/motion evidence, head tilt and head positioning, signal loss, ghosting, or other possible artifacts in the data. Two data were excluded in this step.

#### Preprocessing

The analysis was performed using FEAT (fMRI Expert Analysis Tool), part of FSL (FMRIB’s Software Library, v. 5.0.9). The preprocessing steps included: (1) motion correction using MCFLIRT, FSL (Motion Correction from FMRIB’s Linear Image Registration Tool); (2) skull-stripping for removal of non-brain tissue from the structural T1-weighted images using Brain Extraction Tool (BET), FSL; (3) slice-timing correction (data acquisition: interleaved); (4) spatial smoothing, using a Gaussian kernel of FWHM = 6.0 mm; (5) Melodic ICA data exploration, to identify remaining data artifacts, and to help to better explore activation in the data; (6) multiplicative mean intensity normalization of the volume at each time point; and (7) high-pass temporal filtering (Gaussian-weighted least-squares straight-line fitting, with sigma = 60.0 s). (8) Normalization of the functional images to the standard Montreal Neurological Institute (MNI) brain atlas was also performed *via*: (i) co-registration of the functional images to the high-resolution T1-weighted scan, using FLIRT (FMRIB’s Linear Image Registration) and the BBR (Boundary-Based Registration) cost function; (ii) linear registration of the structural T1 images to the MNI space, with 12 DOF (degrees-of-freedom).

#### First-Level Analysis

The statistical analysis was based on a general linear model (GLM), and was performed using FEAT (version 6.0.0), FSL. The FILM (FMRIB Improved Linear Model) pre-whitening was used for statistical analysis of the fMRI time-series, in order to make the statistical approaches valid and maximally efficient, which devoted a “*z*-score” to the corresponding BOLD signal. As explained above, registration of the estimated function map to the corresponding structural image and ultimately to the MNI space was carried out.

Two types of analysis were performed on the data.

**FIGURE 3:**
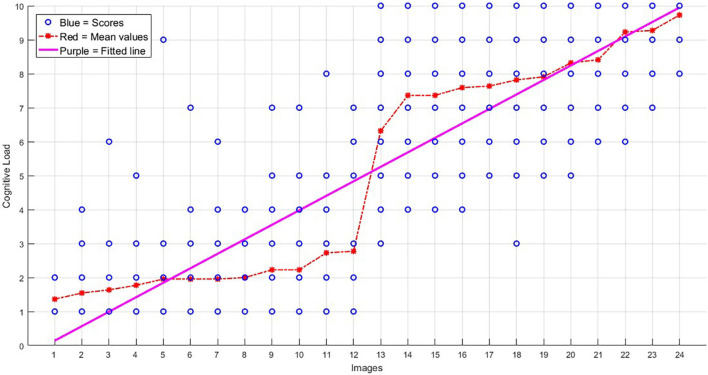
The scores obtained for the 24 images, on their level of cognitive load (images are sorted here from 1 to 24 based on their average load). The blue circles show the scores obtained from the 22 participants of the behavioral group for each image (in most cases, a blue point accumulated the scorings of more than one participant), the red stars show the average scores, and the purple line shows the trend of the increasing load.

(i) Two regressors were defined in the analysis, corresponding to the high and low load conditions. Regarding the responses of the participants during retrieval of the images, those stimuli which received a “weak” score for the success of the retrieval were excluded from the final EVs (explanatory variables) of the GLM analysis. As explained above, the images with a “weak” response were not successfully retrieved. Also, only the portion of the 15-s interval in which the participant was actively recalling the image (before pressing the key) was considered in the EVs. The individual GLM analyses were performed by creating a boxcar function of tasks (different conditions) against rest, being convolved with a canonical hemodynamic response function and its temporal derivatives. Four contrasts were defined here: (a) high load images (average), (b) low load images (average), (c) high minus low, and (d) low minus high contrasts, and we called this analysis a “categorical” analysis.
(ii) The second analysis was performed to identify the possible associations between the degree of the load of the images and brain activity. This analysis was to test if brain activity was modulated by the intensity of the parameter of interest (cognitive load). Parametric study design and data analysis is useful for paradigms with continuous variables (Soch et al., 2021), as we had here. For this aim, a regressor was inserted into the GLM, which included all high- and low-load trials (again excluding the ones with a weak response), with the weight of the trials in the dedicated EV (called parametric EV) being set as the mean cognitive load of that trial, obtained during prior behavioral assessments, and shown by red in Figure 3. The contrast relevant to this EV would obtain brain activations being in association with the cognitive load.

#### Higher-Level Analysis

The group-level analysis was performed using FLAME (FMRIB’s Local Analysis of Mixed Effects) in FSL, to estimate averages for each of the image groups, as well as to compare them. Cluster thresholding was performed only to reveal the significantly-active clusters. The criteria for identification of active clusters (in the average contrast) was a voxel-level probability threshold of *z*-value greater than 3.0; for estimating the contrast of the high and low conditions, and as well as for the parametric analysis, the threshold was set as *z*-value greater than 4.0; False Discovery Rate (*P*_*FDR*_ < 0.05) was used to correct for multiple comparisons.

#### Functional Connectivity Estimation

Following the analysis of activations in brain regions, we performed a functional connectivity analysis on our acquired task-based fMRI data, and between those brain regions which were observed active in our categorical analysis. For this aim, we performed an ROI-to-ROI functional Connectivity analysis using CONN Toolbox (v.18b^1^), and the brain areas provided in Table 3 were regarded as the Regions of Interest (ROI). The ROIs were from the MNI atlas, and were embedded and accessible in the CONN toolbox.

**TABLE 1:** The ID and the age and gender of the included participants.

**Participants ID**	**Age**	**M/F**	**Mental health- DASS tests**			**RAVLT**			**Working memory**	
			**Depression**	**Anxiety**	**Stress**	**A1**	**A2**	**B2**	**FDST**	**BDST**
F-08	29	F	2	0	0	9	14	59	10	5
F-18	39	F	2	3	5	6	14	62	8	6
F-19	37	F	0.5	0	0.5	7	14	57	9	8
F-20	34	F	0	0	0	4 *	14	43	6	6
F-21	32	F	7	5	11	7	13	59	11	9
F-22	40	F	5	2	6	8	15	59	10	8
F-23	38	F	2	0	0	4 *	11	49	9	10
F-25	33	F	2	2	1	9	11	55	8	6
F-26	34	F	3	0	7	8	13	60	5	3 *
F-27	39	F	1	0	0	8	14	57	9	7
F-28	39	F	6	0	3	9	12	59	12	9
F-29	26	F	1	2	3	8	14	58	9	9
F-30	33	F	10	10 *	10	9	14	64	7	8
F-31	21	F	0	2	4	7	11	56	7	8
F-33	30	F	15 *	13 *	14 *	7	13	57	9	5
F-34	21	F	3	2	10	8	12	59	14	11
F-36	38	F	4	3	6	9	13	60	10	8
F-37	25	F	3	2	2	7	14	61	6	8
M-10	22	M	1	1	1	7	11	55	8	8
M-19	26	M	0	2	5	8	12	52	9	7
M-20	24	M	4	0	3	9	9	50	11	9
M-21	21	M	5	0	7	6	11	56	6	5
M-23	26	M	16 *	3	9	6	12	47	8	6
M-24	27	M	4	0	4	6	14	55	6	6
M-25	28	M	3	0	2	6	11	50	14	13
M-26	33	M	0	0	0	8	10	54	12	7
M-28	32	M	1	1	9	8	15	65	10	5
M-31	32	M	0	0	1	8	10	50	9	7
M-32	31	M	2	0	10	9	13	61	7	7

*Using the Depression Anxiety Stress Scales (DASS-21) test, the mental health of the participants was assessed.*

*Three main measures of the Rey Auditory Verbal Learning Test (RAVLT), including A1: Word Span, A2: Delayed Recall, and B2: Total Learning were used to test a healthy episodic memory in the participants.*

*Working memory, was assessed by the Forward and Backward Digit Span tasks.*

*Indicates a score not in the normal range (mean ± 1 SD), based on the normal values reported by Lovibond and Lovibond (1995) (DASS-21), Rezvanfard et al. (2011) (RAVLT), and Orangi et al. (2002) (FDST and BDST).

**TABLE 2:** The brain areas with a significant activation when retrieving high load and low load images; The *z*-value, and the coordinates (x,y,z) in the MNI space are provided for each brain structure.

**High load images- average**						**Low load images- average**					
**Cluster**	**Voxels**	***Z*-max**	**X**	**Y**	**Z**	**Cluster**	**Voxels**	***Z*-max**	**X**	**Y**	**Z**
5	26,946	7.67	−4	16	44	9	9,764	7.4	−2	20	44
4	4,165	6.93	−24	−64	54	8	4,417	6.93	30	−60	−28
3	604	4.68	−38	56	12	7	3,806	7.12	−28	−60	50
2	428	5.81	34	20	6	6	3,151	7.44	−12	8	6
1	276	3.88	46	34	24	5	2,682	6.47	−44	−64	−8
						4	2,352	6.01	32	−52	46
						3	820	6.7	34	20	6
						2	615	4.73	48	34	26
						1	435	6.23	0	−46	−22

**Brain structures**	**High load contrast**		**Low load contrast**	
	**Z/x,y,z (L)**	**Z/x,y,z (R)**	**Z/x,y,z (L)**	**Z/x,y,z (R)**

Paracingulate	7.67/−4,16,44	5.4/4,16,42	7.4/−2,20,44	6.9/8,10,48
Parahippocamp	4.5/−26,−34,−16	7.0/30,−34,−16	3.4/−32,−38,−12	3.4/34,−36,−12
Cerebellum	5.5/−32,−56,−30	6.8/8,−78,−20	6.0/−28,−62,−26	6.9/30,−60,−28
Sup. Lat. Occ.	6.9/−24,−64,54	4.4/24,−66,54	7.1/−28,−60,50	5.9/32,−62,46
Sup. Par.	6.4/−36,−48,46	4.4/34,−46,46	6.8/−34,−50,44	6.0/32,−52,46
Frontal pole	4.7/−38,56,12	3.6/40,38,18	4.3/−32,52,20	4.4/46,38,20
Insula	6.5/−30,22,6	5.8/34,20,6	6.9/−30,22,4	6.7/34,20,6
MFG	5.4/−48,14,32	3.9/46,34,24	6.5/−38,30,20	4.7/48,34,26
Precentral	4.5/−48,2,32	3.3/38,−2,60	5.4/−48,4,32	6.2/42,6,32
ACC	4.4/−4,12,40	4.4/8,20,32	–	–
Sup. Fron.	4.4/−16,0,70	3.4/10,0,70	3.3/−16,6,64	3.4/14,4,64
SMA	6.5/−4,4,58	5.4/4,4,58	4.3/−2,0,64	3.4/6,2,64
Caudate	5.6/−16,2,18	4.5/18,4,18	7.4/−12,8,6	6.0/16,0,16
Precuneus	4.5/−20,−60,18	5.5/20,−60,18	–	–
Thalamus	4.5/−2,−4,4	3.5/4,−4,4	3.4/−2,−4,4	3.5/10,−4,4
IFG	4.4/−46,8,26	3.4/38,10,26	3.4/−52,14,2	3.71/54,22,2
Fusiform	6.5/−32,−40,−14	5.5/30,−40,−14	3.4/−30,−50,−14	4.4/30,−40,−14
Lingual G.	3.4/−8,−80,−14	4.4/6,−82,−14	3.4/−6,−76,−14	3.4/10,−78,−14
Hippocampus	–	3.4/18,−18,−16	–	–
Pallidum	6.6/−16,2,2	5.6/16,4,2	6.2/−18,0,2	6.7/14,4,2
Inf. Lat. Occ.	5.5/−50,−66,−8	–	6.5/−44,−64,−8	4.4/52,−62,−8
Inf. Temp.	6.6/−54,−62,−12	5.5/50,−56,−12	6.4/−52,−62,−12	5.4/54,−56,−12
Mid. Temp.	3.4/−56,−58,−6	–	5.9/−52,−58,−6	4.4/54,−52,−6

*Sup, superior; Lat, lateral; Occ, occipital; Par, parietal; MFG, middle frontal gyrus; ACC, anterior cingulate cortex; Fron, frontal; SMA, supplementary motor area; IFG, inferior frontal gyrus; G, gyrus; Inf, inferior; Temp, temporal; Mid, middle.*

**TABLE 3:** The brain activations relevant to the “high load > Low load” and the “high load < low load” contrasts; The brain activations observed during the parametric analysis; The *z*-value, and the coordinates (x,y,z) in the MNI space are provided for each brain structure.

**“High > Low” contrast**						**“High < Low” contrast**						**“Parametric” contrast**
**Cluster**	**Voxels**	***Z*-max**	**X**	**Y**	**Z**	**Cluster**	**Voxels**	***Z*-max**	**X**	**Y**	**Z**	
10	1,625	6.97	18	−58	22	17	865	5.45	44	−46	60	
9	674	5.99	−34	−80	30	16	817	5.39	−60	−32	48	
8	548	6.05	−26	−44	−8	15	618	5.59	46	42	8	
7	507	5.82	−12	−64	48	14	234	5.48	58	−58	−14	
6	437	5.7	30	−34	−16	13	186	5.37	50	12	34	
5	269	5.41	40	−80	34	12	164	5.37	4	−22	44	
4	51	5.2	14	−82	−12	11	135	5.1	−38	−2	8	
3	35	5.23	−16	−40	−42	10	109	5.24	6	42	40	
2	35	4.94	2	−86	−2	9	97	5.15	64	−28	−10	
1	24	5.18	4	−50	−32	8	96	5.5	42	2	14	
						7	74	5.72	−6	0	40	
						6	56	5.11	−54	−68	−6	
						5	33	5.07	−32	20	−**12**	
						4	29	5.22	54	10	−16	
						3	28	4.78	30	24	−10	
						2	22	4.94	−58	−22	20	
						1	21	5.05	−54	6	30	

**Brain structures**	**“High > Low contrast”**		**“High < Low” contrast**		***Z*-value L/R**
	**Z/x,y,z (L)**	**Z/x,y,z (R)**	**Z/x,y,z (L)**	**Z/x,y,z (R)**	

Parahippocamp	5.6/−14,−36,−10	5.7/30,−34,−16	–	–	5.6/5.8
Cerebellum	5.2/−16,−40,−42	5.2/4,−50,−32	–	–	None/5.2
Sup. Lat. Occ.	5.9/−34,−80,30	5.4/40,−80,34	–	–	–
Fusiform	–	4.4/24,−84,−8	–	–	5.1/5.5
Lingual G.	6.1/−26,−44,−8	5.5/20,−38,−10	–	–	5.9/5.4
Precuneus	6.4/−12,−54,10	6.9/18,−58,22	–	–	6.2/7.1
PCC	5.8/−8,−48,4	6.5/8,−48,8	–	–	6.0/6.5
Precentral	–	–	5.1/−54,6,30	5.2/58,10,26	–
Sup. Fron.	–	–	–	5.2/6,42,40	–
IFG	–	–	–	5.5/56,20,−2	–
SMA	–	–	4.9/−4,−14,52	4.7/2,−10,48	–
ACC	–	–	5.7/−6,0,40	4.3/6,−2,40	–
Frontal pole	–	–	–	5.6/46,42,8	–
Insula	–	–	5.1/−38,−2,8	5.2/36,4,14	–
Inf. Temp.	–	–	–	5.5/58,−58,−14	–
Mid. Temp.	–	–	–	5.3/64,−48,−10	–
Supramarginal G.	–	–	5.4/−60,−32,48	5.4/64,−24,40	–
Postcentral G.	–	–	5.3/−44,−28,42	–	–
Paracingulate	–	–	–	5.1/8,38,34	–
Sup. Par.	–	–	–	5.5/44,−46,60	–
Middle Fron.	–	–	–	5.4/50,12,34	–

*Sup, superior; Lat, lateral; Occ, occipital; Par, parietal; MFG, middle frontal gyrus; ACC, anterior cingulate cortex; Fron, frontal; SMA, supplementary motor area; IFG, inferior frontal gyrus; G, gyrus; Inf, inferior; Temp, temporal; Mid, middle.*

For the sake of preprocessing the images, functional images were first subject to motion estimation and correction, then translation to center (0, 0, 0 coordinates), slice timing correction, ART-based (Artifact Detection Tools) identification of outlier scans^2^, tissue segmentation, normalization to MNI space, and spatial smoothing with a Gaussian kernel (8 mm FWHM). The structural images were also centered, segmented and normalized to the MNI space.

A weighted-GLM (general linear model) was then used to conduct first-level analysis. The functional connectivity of the ROIs were estimated using three groups of regressors: (I) mean of the high and low load conditions; (II) the high and low load conditions vs. rest; and (III) high vs. low load condition. The connectivity estimation was based on the bi-variate correlation method, and using HRF-weighting. Prior to this step and in order to reduce the effect of noise, the resulting preprocessed images were band-passed filtered to 0–0.1 Hz; the effect of denoising was visualized here by illustrating the mean of the distribution of connectivity values for each subject. For the second-level analysis of connectivity, and for between-conditions distinction, F-statistic test was used, with the significancy level based on *P*-value < 0.001 (FDR corrected). In order to validate the multiple comparisons, significance tests were based on standardized *Z*-scores.

## Results

### Mental Health Assessments

The 21-item version of the questionnaire on depression, anxiety, and stress scale (DASS-21) was used as a quantitative measure of distress. The average scores were 3.53 ± 4.06 (mean ± standard deviation) for depression, 1.83 ± 3.01 for anxiety, and 4.60 ± 4.02 for stress. In Table 1, the scores of the DASS-21 test for each individual are also provided, and the individuals who met the mental health criteria (Lovibond and Lovibond, 1995) are indicated by asterisk.

### Cognitive Assessments

#### RAVLT

The average scores for the 11 sections of the RAVLT were as follows. (A1) Word Span: 7.41 ± 1.4; (A2) Delayed Recall: 12.55 ± 1.61; (A3) Proactive Interference Score: 1.48 ± 1.59; (A4) Retroactive Interference Score: 0.79 ± 1.49; (A5) Forgetting Rate: 0.48 ± 1.35; (A6) Position Effect: 0.34 ± 2.89; (B1) Final Acquisition Learning: 13.55 ± 1.02; (B2) Total Learning: 56.17 ± 5.15; (B3) Learning Over Trials: 19.10 ± 6.03; (C1) Net Positive Score: 14.31 ± 0.96; (C2) Recognition Over Recall: 1.82 ± 1.46. The scores of each individual in this test are provided in Table 1, and based on the normative values reported for the young Iranian population (e.g., A1 = 7.03 ± 2.43; A2 = 11.27 ± 2.57; B2 = 52.57 ± 9.74) (Rezvanfard et al., 2011), the individuals with a normal performance are indicated.

#### Forward/Backward Digit Span Task

For the forward DST, the average length of the chain of recalled digits was 5.93 ± 1.22, and the average score here (number of correct trials) was 9.10 ± 2.41. In the backward DST, the average length of the chain of recalled digits in a reversed order was 5.59 ± 1.40, with the average score of 7.38 ± 2.04. We indicated in Table 1 the normal WM performance of the participants based on their individual scores, and due to the criteria which were reported previously for the Iranian population (Orangi et al., 2002).

### Behavioral Assessments

Based on the behavioral assessments of the cognitive load of the images, obtained from the behavioral group, the mean score of the “low load” images was 2.01 ± 1.44, whereas this score was 8.07 ± 1.81 for the “high load” images. The scores of the two groups of images were statistically significantly different (*p*-value < 1.0e-5).

Figure 3 shows the behavioral scores obtained for the 24 images. As is shown, the mean scores of the low load images (images 1–12) are significantly different from the high load images (images 13–24), and also there is an increment trend in the load of the 24 images.

### Retrieval Performance

**FIGURE 4:**
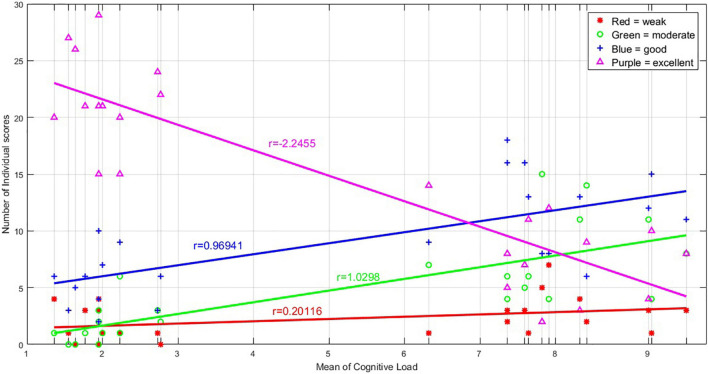
The graph shows the number of excellent, good, moderate, and weak responses obtained for any of the 24 images, during the retrieval phase in the MR scanner. There are 24 vertical lines corresponding to the 24 images sorted based on their average cognitive load, and on each vertical line, the number of excellent, good, moderate, and weak responses to each image are illustrated. The number of excellent responses declined with the increasing load of the images, and therefore, the number of good and moderate response increased; the number of weak responses seems unchanged.

Figure 4 shows the performance of the participants when retrieving the images. As described before, each individual had to rate him/herself on the quality of recalling the details of the images as weak, moderate, good, or excellent. Based on the results, 47.6% of the responses were excellent, 28.8% were good, 15.4% were moderate, and only 7.2% were weak. In addition, by increasing the load of the images, the frequency of “excellent” responses dropped (linear rate = −2.2), and as a result, the rates of moderate (*r* = 1.0) and good (*r* = 0.96) responses increased.

### Retrieval Duration

**FIGURE 5:**
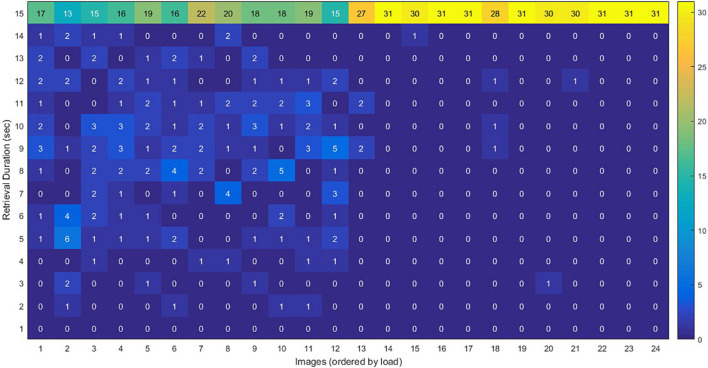
The distribution of the retrieval duration for each of the 24 images, during the fMRI scan. The vertical axis shows the amount of time for retrieving an image by each participants (the numbers in each cell represent the number of participants). When the load increased, the number of people who took a longer time for retrieving an image increased. For example, 17 of the participants used the whole duration (15 s) for retrieving image 1, whereas all participants (#31) used the whole duration for retrieving image 24.

Figure 5 shows the distribution of the retrieval duration for each of the 24 images. As is illustrated, by increasing the load of the images, the number of participants who did use the whole duration of the retrieval (15 s) was also increased.

### Load Effect on Brain Activations

In the first analysis of fMRI data, we contrasted the brain activations relevant to retrieving high load images vs. low loads; as mentioned above, the images with a “weak” response were excluded from this analysis. Five clusters of activation were observed in average brain activations relevant to retrieving high load images, and nine clusters for the low load. As provided in detail in Table 2, 23 brain structures were observed active in the high load contrast, including paracingulate, parahippocampus, cerebellum, superior lateral occipital, superior parietal, and eighteen other brain areas. Among these areas, only the ACC, precuneus, and hippocampus were not observed active in the low load contrast. This analysis was performed with a *z*-value greater than 3.0.

**FIGURE 6:**
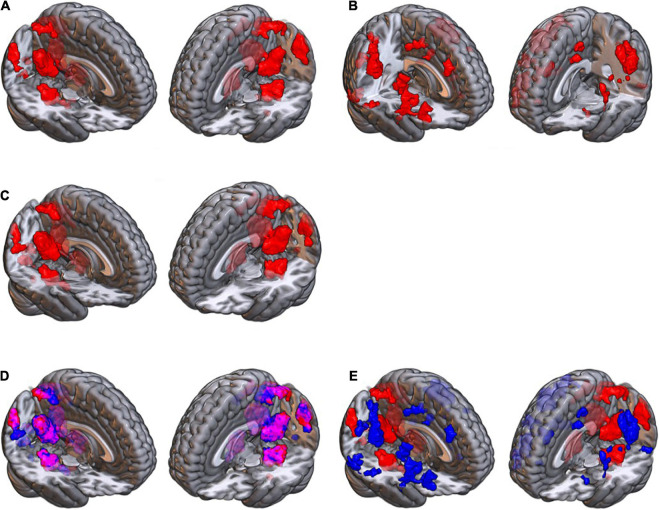
The brain maps obtained for **(A)** the high load greater than low load contrast; and **(B)** the high load smaller than low load contrast, using the categorical data analysis; **(C)** the brain areas with an association with cognitive load, in the parametric analysis; **(D)** the overlap of the high minus low (red) map and the parametric (blue) contrast map, which illustrates similarity of the brain areas between these two contrasts (illustrated in purple color); **(E)** the overlap of the high minus low (red) map and low minus high (blue) contrast map, which shows there is no similar areas between these two (no purple color voxel).

The next step was to contrast the brain activations for the two load levels. Seven brain structures showed a higher activation when retrieving high load images, compared to low; these areas included bilateral parahippocampus, bilateral cerebellum, bilateral superior lateral occipital, right fusiform gyrus, bilateral lingual gyrus, bilateral precuneus, and bilateral posterior cingulate cortex. On the other hand, fourteen structures had a lower activation when retrieving high load images, including bilateral precentral gyrus, right inferior and superior frontal gyri, bilateral SMA, and ten other brain areas. Details of these activations are provided in Table 3, and the areas are illustrated in Figures 6A,B.

### Parametric Analysis

The third analysis was a parametric analysis in which we tested the association of the cognitive load of the images with the brain activations using a “parametric” contrast. As illustrated in Figure 6C and provided in Table 3, when retrieving images, the activation of six brain structures showed associations with the cognitive load of the images in the parametric contrast, including precuneus, posterior cingulate cortex, lingual gyrus, parahippocampus, fusiform gyrus, and cerebellum. As illustrated in Figure 6D, these areas had a considerable overlap with the brain map obtained for the “high load minus low load” contrast. On the contrary, the overlap of the “high minus low load” and the “low minus high load” maps, Figure 6E, showed that there was no similar brain areas between these two maps.

### Functional Connectivity Estimates

**FIGURE 7:**
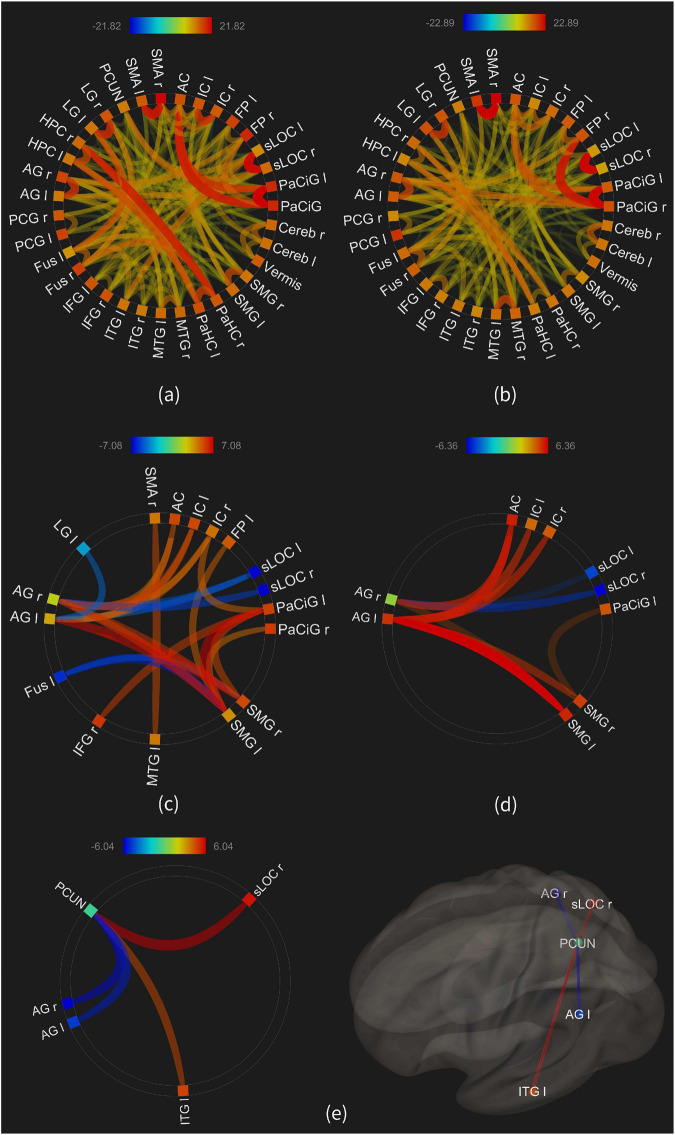
The functional connectivity of the 35 ROIs; **(a)** average connectivity in the “high load” condition; **(b)** average connectivity in the “low load” condition; **(c)** connectivities in the “high minus rest” condition; **(d)** connectivities in the “low minus rest” condition; **(e)** connectivities in the “high minus low” condition.

The aim here was to investigate how the functional connectivity between the active brain areas would change with the retrieval load. Thus, the FC was estimated in three groups of contrasts as described in Methods, and the results are illustrated in Figure 7. This figure only shows the connectivities above the significance level (*p*-FDR < 0.001) including both the positive and negative connectivity estimates, and the thickness and transparency of each line in the Figure is proportional to the connectivity estimates.

Figures 7A,B show the average connectivity of the ROIs in the high and low load conditions, respectively. As illustrated, large number of connections are observed, such as between the parahippocampus and hippocampus in the high load, or between the frontal pole and paracingulate in the low load. Visual comparison of the two maps shows larger number and more significant connectivities in the high load condition, compared to the low load.

Figures 7C,D show the connectivities in the “high minus rest” and “low minus rest” conditions, with seventeen ROIs in the first and ten ROIs in the second condition showing a significant connectivity. This result, similar to the above, shows an increased number and strength of functional connectivity between the brain areas when retrieving the images with a higher load, compared to the low load.

Figure 7E shows the results of a statistical comparison between the connectivities in the high and low load conditions. The FC between the precuneus and the right superior lateral occipital was increased in the high load [beta = 0.2; *T*-value(30) = 6.04, *p*-FDR < 0.00004], as well as between the precuneus and the left inferior temporal gyrus [beta = 0.16; *T*-value(30) = 4.74; *p*-FDR < 0.0004]. On the other hand, the connectivity between the precuneus and both the right [beta = −0.21; *T*-value(30) = −5.72; *p*-FDR < 0.00005] and left [beta = −0.17; *T*-value(30) = −5.33; *p*-FDR < 0.0001] angular gyri was lower in the high load condition.

## Discussion

### Review of the Results

In this study we aimed to investigate how changing the cognitive load during LTM retrieval would modulate brain activations. This question was tested using fMRI and by using two different analysis methods. We performed a number of mental and cognitive assessments to assure that our participants had a healthy brain and a normal memory ability; as illustrated in Table 1, two of our participants showed deviations from the normal range in their mental health results; in a control analysis, we observed that removing those two data from our dataset did not significantly alter our findings and conclusions.

With increasing load, the quality of remembering the images declined, and the participants used a longer time to recall the images (Figure 5). Seven brain structures showed a higher activation when retrieving higher load images, and a number of brain structures also showed a reduced activation in the higher load (Table 3), which will be discussed below.

The participants reviewed the images for 12 days, until 2 days before their fMRI scan. The 2 days were considered for the consolidation phase, referred to as stabilization of memory (McGaugh, 2000). During this stage, the labile memory becomes stable and resilient to interferences (Santini et al., 2014). The Standard Consolidation Theory (SCT) proposes that after consolidation, the memory will be represented in a distributed cortical network (Winocur et al., 2010). Consolidation is suggested to happen during sleep (van der Helm et al., 2011; Weber et al., 2014), conceptualized as “active system consolidation” (Vorster and Born, 2015).

Our participants had 15 s to retrieve the details of the memorized images. Studies have shown that the retrieval happens in a much shorter time; access to word meaning occurs in a few hundred milliseconds (Neely, 1991), image generation of common items takes on average about 1 s, and access to factual knowledge takes about 1,200 ms (Kane et al., 2007). Recalling autobiographical memory may take 2–3 s (Conway, 2005) or up to 5–7 s (Conway, 2005). Nearly all our participants used their 15-s time for recalling the high load images, whereas fewer people used the whole duration for the low load images (Figure 5), suggesting the appropriate selection of the 15-s interval.

### Retrieval Performance

In our results, the number of “excellent” responses during imaging declined in higher load, which shows the retrieval performance decrement (Figure 4). There are similar previous findings, particularly on the WM, including adverse association of performance on the anti-saccade task with the cognitive load (Rissman et al., 2009), impairments in the performance of the go/no-go task in higher WM load (Hester and Garavan, 2005), performance deterioration in higher memory load (Leung et al., 2005), decrement of accuracy by increasing WM load (Intaitė et al., 2016), and accuracy decrement as the digit load increased (Ma et al., 2012).

The deterioration of performance is explained previously by a lower accuracy or by a longer reaction time (Leung et al., 2005). We observed that retrieving the higher load images on average took longer (Figure 5), and although our behavioral assessments during the fMRI scan had methodological limitations in terms of the temporal resolution, previous studies with more robust methods have also reported an increase of reaction time with increasing load (Zysset et al., 2001; Kirschen et al., 2005; Axmacher et al., 2009; Solman et al., 2011; Bennett et al., 2013). The association of cognitive load with the reaction time could possibly be explained by the serial or parallel information processing in brain.

Serial processing means sequential; the processing of each object takes the same amount of time, and the next processing begins when the previous one is finalized (Townsend, 1990). On the contrary, parallel processing means simultaneous processing on several objects. In a study on reaction time, Sternberg (1966) showed that their findings could only result from a serial processing in the brain. Another study, by the simultaneous presentation of two tasks, also showed that cognitive control processes are serially allocated to the tasks (Cocchi et al., 2011). However, parallel models are also suggested, such as by the study which explained the linear RT (response time) curves (Atkinson et al., 1969; Townsend and Fifić, 2004). Some investigators have suggested that short ISIs (inter stimulus interval) may be more associated with parallel processing, whereas longer ISIs more result in serial processing (Forrin and Cunningham, 1973). Our results showed an increment in brain activations as well as a longer response time in the higher load (Table 3 and Figure 5), which may be suggestive of both parallel and serial information processing. As stated previously, both the serial and parallel information processing are simultaneously recruited in the brain (Townsend and Fifić, 2004), and therefore identifying the method which is more utilized in the higher load needs further studies.

### Association of Cognitive Load With Brain Function

Our results showed changes in brain function in association with an increasing load in LTM retrieval (Table 3). Previous works also showed changes in the pattern of brain activity with an altered cognitive load, such as an increase in the theta band power in a higher WM load (Ma et al., 2012), or changes in the lateralization of brain function (Kirschen et al., 2005). There are explanations for the brain activity increment in response to an increasing load; examples include a higher computational demand in the regions involved in that function (Leung and Alain, 2011), the brain regions to track task difficulty and therefore working harder to perform a more difficult task (Gould et al., 2003), or utilizing a higher-level cognitive control process in a higher load (Fegen et al., 2015).

A number of models are also proposed to explain the effect of cognitive load on brain function. (i) The first model explains the mental fatigue associated with the cognitive demand (Ishii et al., 2014b). In this model, the load activates two systems: a system to maintain performance against fatigue, whose activation increases with motivation (called facilitation), and an inhibition system which impairs the performance (inhibition). Depending on the balance between the two systems, the performance may be impaired, maintained, or even improved. (ii) The second model is called CRUNCH (Compensation-Related Utilization of Neural Circuits Hypothesis) (Reuter-Lorenz and Cappell, 2008), which suggests that the over-recruitment of neural resources to maintain the performance in a high load condition has a cost, which is the exhaustion of the neural sources in the subsequent tasks; and (iii) as suggested previously (Zysset et al., 2001), in more difficult tasks, either a different retrieval process and network or a highly adaptive process is used, in which the neural activity of brain regions change in accordance to task difficulty. Since in our study the high and low load images were randomly distributed, the first two models could not explain our findings, and the latter model is more likely.

#### Increased Activation

Seven brain structures showed an elevated activation during the higher load of LTM retrieval in our work, including bilateral parahippocampus, bilateral cerebellum, bilateral superior lateral occipital, right fusiform gyrus, bilateral lingual gyrus, bilateral precuneus, and bilateral posterior cingulate cortex (Table 3). A study has suggested that if a brain area is crucial for memory, its activity should be modulated by memory load (Leung et al., 2005). This notion was approved in WM (Cook et al., 2007), and our findings also showed this for the LTM.

The precuneus showed an increased activation in the higher load in our work. This brain structure is illustrated previously to be involved in a number of brain functions which were vital for our fMRI task, including mental imagery (Daselaar et al., 2008; Huijbers et al., 2011), storage of information (Hartley and Speer, 2000), mental representation of space (Bueti and Walsh, 2009), and visual imagery during episodic retrieval (Cavanna and Trimble, 2006). In addition, the activation of this region was observed to be greater during imagined than the viewed pictures (Lundstrom et al., 2003), and was also associated with the period a memory was maintained in the brain (Daselaar et al., 2008), which all justify the observed pattern of precuneus activation.

The occipital brain areas also showed a higher activation in the higher load (Table 3). It is reported that elaboration of memory involves an imagery function evoked by the retrieved episode, particularly in the visual system (Rubin, 2006), and the occipital areas were active in providing visual imagery when people remembered what visual scenes looked like (Raz and Levin, 2014). The posterior brain regions, and particularly the visual areas, are involved in memories with a high degree of subjective reliving, and studies have shown it as the best predictor of the degree of experienced reliving in visual imagery (Rubin, 2006). In our results, we observed the visual cortex to be active when there was no visual stimulation (participants had their eyes closed); this is in accordance with the studies which showed that the sensory regions that are active during encoding are reactivated during retrieval (Prince et al., 2005).

Cerebellum was also more active in the higher load (Table 3), which could be explained by its role in sequential information processing (Tedesco et al., 2011), and in spatial memory (Kirschen et al., 2005). This region was traditionally thought to be only involved in motor control and balance, but its role in cognition is now considerable (Kirschen et al., 2005). The increased activation we observed in the cerebellum was previously illustrated in the high load of auditory WM as well (Leung and Alain, 2011), which is suggested to be a domain-general activation to respond to the increased demand for sensory memory (Petacchi et al., 2005), increased involvement of working memory (Kirschen et al., 2005), or increased activity in the cerebro-cerebellar networks for optimizing functions (Salmi et al., 2009).

Similarly and in our parametric analyses, six brain areas showed an increased activation in the parametric analysis (Table 3), which was similar to the results of previous works on the cerebellum (Cairo et al., 2004; O’Hare et al., 2008), parahippocampus, posterior cingulate, and precuneus (Kirschen et al., 2005). A suggested reason for such results is the involvement of PCC in many kinds of self-monitoring processes such as the level of fatigue sensation (Cook et al., 2007), or the parahippocampus being a locus of the WM-LTM interaction (Axmacher et al., 2009). It is suggested that the linear activation increase in a brain region is related to the processing demands (Leung et al., 2005), such as what happens to complete a task such as retrieval (Gould et al., 2003).

One of the few similar studies (Zysset et al., 2001) available revealed that with increasing task difficulty, no additional brain regions became involved in LTM retrieval, but a highly adaptive process was involved during the load alteration. As provided in our results in Table 2, the brain regions involved in the retrieval of low load and high load images do highly overlap; among the 23 structures, only three regions were not observed active in the low load. This pattern stabilizes the old finding on the involvement of an adaptive process during LTM retrieval, compared to acquiring additional brain areas to manage the increasing load.

#### Declined Activation

A number of brain regions showed a declined activation when retrieving higher load images, as detailed in Table 3. There are mechanisms suggested for that.

(i) The task-induced deactivation, which is the declined activation of brain regions in the demanding tasks, is suggested previously (Rissman et al., 2009); in response to increasing load, the attentional and perceptual resources decline (Cocchi et al., 2011), and it can for example result in a weaker encoding of the stimulus in the high load (Lavie, 2006). In another work, the high load prevented the encoding of irrelevant information (Addis et al., 2004), or task-irrelevant stimuli (SanMiguel et al., 2008). The brain regions that we observed with a declined activation are involved in important functions; however, they may not be vital for LTM retrieval, and therefore the resources are taken away from these regions, and are allocated to more responsible brain areas. As an example, prefrontal underactivation was observed when resources were limited due to task complexity (Nagel et al., 2009; Cappell et al., 2010). The resource allocation alteration improves the performance, for example in increasing the number of items an individual could memorize (Linden et al., 2003), or for better solving difficult math problems (MacNamara et al., 2012).
(ii) A different retrieval mechanism may be recruited in higher load; a suggestion was the retrieval process relying less on the short-term memory but more on the direct retrieval/scanning processes (Zysset et al., 2001). In short lists, the items were kept in short-term memory to be scanned, whereas for longer lists the items are retrieved from LTM and scanned, without being temporally stored in STM.
(iii) With increasing load, some brain regions show a non-linear response toward that; an example is the inverted U-shape pattern observed in the precentral, superior frontal, and the SMA areas in higher WM load (Linden et al., 2003), or the non-linear behavior observed in IFG (Gould et al., 2003; Ishii et al., 2014a). Because non-linearity is not assessed here, some of the linear activation increases here may have a “capacity constrained” (inverted-U) shape, with a decreasing signal change in higher memory load (Gould et al., 2003). A non-linear trend may also indicate engagement of different memory mechanisms at different load levels (Braver et al., 1997).
(iv) Studies which show a decreased activation in response to greater cognitive load are interpreted as attention being allocated away from cognitive functions performed by default network regions (Binder, 2012) or a reduction of internal thoughts (Andrews-Hanna, 2012). Load-dependent decreases in the activation of default network regions are observed (Huang et al., 2016). Limited attentional resources are easily exhausted by the demanding tasks, which leaves little attention for further processing, resulting in attentional gating (Lavie, 2006). The load could also reduce the processing of salient stimuli by altering the attention (MacNamara et al., 2012).

As stated above, load-dependent decreases in the activation of the default network regions of the brain is noted; however, as observed in our results, some areas of the default mode network (DMN) were active in the higher load in our study; this finding should be discussed.

The DMN is a brain network that shows higher activity in resting state, compared to the goal-oriented tasks (Jeong et al., 2015), and it includes a network of brain regions, including the mPFC, precuneus, PCC, IPL, lateral temporal cortex, and hippocampal formation (Raichle et al., 2001; Buckner et al., 2008; Mohan et al., 2016). There are reports on the deactivation of this network upon initiation of a goal-directed behavior (Mak et al., 2016), or when executive functions were required (Buckner et al., 2008). The DMN suppression will also increase with task difficulty, suggesting that introspective attentional resources must be reallocated to focus on an extrinsic task (Singh and Fawcett, 2008). In other words, this network reconfigures itself in a demand specific fashion to support behavior (Hasson et al., 2009).

Nonetheless, the areas of the brain are not only involved in one single functional network, and there are reports on the activation of a brain region in multiple functions. As an example, mPFC is observed to be involved in schema processing, social cognition (Schilbach et al., 2008) and affective processing (Roy et al., 2012), or lateral temporal regions have been noted to be involved in autobiographical memory (Svoboda et al., 2006), theory of mind (Gallagher and Frith, 2003), default mode (Shulman et al., 1997), and have been implicated in prospection as well (Schacter et al., 2008). As a result, it is suggested that there is a strong overlap between the brain areas involved in resting state networks and in task dynamics (Smith et al., 2009).

Based on that, numerous studies have illustrated the involvement of some of the brain regions of the DMN in the memory function; examples include activation of the DMN when retrieving a past experience (Daselaar et al., 2009), the mPFC being a core hub of the DMN and being involved in memory processing, reconfiguration of the DMN supporting the schema memory, decoupling of the parahippocampal gyrus of the DMN to facilitate episodic memory (Müller et al., 2020), activity of the DMN being linked to mind wandering which depends on recalling memories (Mason et al., 2007), DMN network connectivity being in relation to episodic memory retrieval and future imaging (Bellana et al., 2016), activation of the parahippocampal gyrus as a primary hub of the DMN predicting memory (Ward et al., 2014b), modulation of DMN coupling to medial temporal regions being associated to episodic memory retrieval (Bellana et al., 2016), DMN supporting memory functioning (Staffaroni et al., 2018), disruptions in the default-mode network being associated with memory performance deficits (Ward et al., 2014a), and the areas of the DMN including parahippocampal gyrus, posterior cingulate cortex/precuneus, inferior parietal lobule, and medial prefrontal cortex being active during immediate recall and delayed recall (Huo et al., 2018).

It is also shown that autobiographical memory activates rather than de-activate some areas of the default mode network (Buckner et al., 2008), and the same pattern has been observed for imagining the future (Schacter et al., 2007), making judgments about oneself and others (Van der Meer et al., 2009), making moral judgments (Boccia et al., 2017), and engaging in theory of mind-type reasoning (Schurz et al., 2014). As a result, the involvement of some areas of the DMN network when retrieving the higher load images in our results could be due to the role of DMN in the memory process, as suggested above.

### Functional Connectivity

As illustrated in Figure 7, the left inferior temporal gyrus and the right superior lateral occipital cortex showed a stronger FC with the precuneus in the higher load, whereas the right and left angular gyri showed a declined FC with precuneus in the same condition. The precuneus showed a central role in the alteration of functional connectivity between the brain areas with an altering load. One reason could be its involvement in key brain functions, such as recollection, cue reactivity, mental imagery strategies, and episodic memory retrieval (Borsook et al., 2015).

In addition, the brain regions which were functionally connected to the precuneus are also shown to be involved in the functions necessary for our fMRI task. For example, LOC plays role in object recognition (Grill-Spector et al., 2001) and also in face perception (Nagy and Turecki, 2012), or the ITG, a part of “what” pathway, is responsible in recognizing objects specially from their form and color, and is also involved in memory retrieval in order to identify the object.

It seems one mechanism for retrieving scenes with much higher details and colors is a stronger connectivity between the brain areas with similar functions. In a study on episodic memory and albeit at the encoding level, an altered (increased) functional connectivity was observed between the left hippocampus and the bilateral ventrolateral prefrontal cortex and the right temporo-parietal junction in a deep compared to shallow encoding (Schott et al., 2013). Alteration of the functional connectivity between the brain areas due to an altering cognitive load is also observed, such as between the IFG, fusiform, and hippocampus (Rissman et al., 2008), and these load-dependent changes of connectivity suggest that these neural circuits dynamically trade-off to accommodate the particular demands of the task (Rissman et al., 2008).

On the contrary, the bilateral angular gyrus showed a declined connectivity with the precuneus in the higher load (Figure 7). This region is part of the “*core network”* which is associated with episodic simulation and episodic memory (Thakral et al., 2017). Research shows that angular gyrus’ dysfunction results in impaired episodic memory in some aspects (Bonnici et al., 2018), and therefore future studies are needed to identify the exact mechanism through which the declined FC of this brain area with the precuneus is observed during the higher load.

### Limitations

Despite our endeavor to select robust methods for our study, there are a few limitations in this work. There are associations between WM and LTM, and although we asked our participants to stop reviewing the images as soon as there was no new information to recall, there is a possibility that WM was also involved in our results. Our parametric analysis only tested the linear associations, whereas a non-linear trend would also be possible in our results. Besides, performing a parametric analysis which investigates the association of brain activations with the “cognitive load” scores that the individuals who underwent the fMRI scan devoted to each image would also be informative. We also were not able to check the amount of details an individual recalled when retrieving an image, and only relied on their subjective reports, which should be considered in the future works. And finally, from activation alone, this is not possible to say that a brain region was the site of storage, processing, or retrieval of the memory, and therefore more complicated study designs are needed for such a distinction.

## Data Availability Statement

The raw data supporting the conclusions of this article will be made available by the authors, without undue reservation.

## Ethics Statement

The studies involving human participants were reviewed and approved by the National Institute for Medical Research Development (NIMAD). The patients/participants provided their written informed consent to participate in this study.

## Author Contributions

MS collected the MRI data, performed cognitive assessments, analyzed all MRI data, prepared the figures and tables of the work, and prepared the methods and results the manuscript. PS supervised the project, developed the idea, helped in fMRI task design, suggested the cognitive tests, and final revision of manuscript. SB had the initial idea of the work, financially supported the work, supervised the project, prepared the manuscript, and revised the manuscript. All authors contributed to the article and approved the submitted version.

## Conflict of Interest

The authors declare that the research was conducted in the absence of any commercial or financial relationships that could be construed as a potential conflict of interest.

## Publisher’s Note

All claims expressed in this article are solely those of the authors and do not necessarily represent those of their affiliated organizations, or those of the publisher, the editors and the reviewers. Any product that may be evaluated in this article, or claim that may be made by its manufacturer, is not guaranteed or endorsed by the publisher.

## Footnotes

1
https://www.nitrc.org/projects/conn

2
https://www.nitrc.org/projects/artifact_detect/
